# Supplementary material for: Novel Anthraquinone Compounds Inhibit Colon Cancer Cell Proliferation via the Reactive Oxygen Species/JNK Pathway
Source: Molecules. 2020 Apr 4;25(7):1672. doi: 10.3390/molecules25071672 (PMC7180728; doi:10.3390/molecules25071672)
Supplement: Supplementary file 1 [file molecules-25-01672-s001.pdf]

Supporting Information

Novel Anthraquinone Compound Inhibits Cancer Cell Proliferation Via the Reactive Oxygen Species/JNK Pathway

Yuying Li*, Fang Guo, Tinggui Chen, Kaiqing Ma, Liwei Zhang, Zhuanhua Wang, Qiang Su, Liheng Feng, Yaoming Liu, Yuzhi Zhou

Key Laboratory for Chemical Biology and Molecular Engineering of Ministry of Education, Institute of Biotechnology, Shanxi University,Taiyuan, 030006, China

*Corresponding author.

E-mail address: lyy9030@sxu.edu.cn(Y. Li)

Table of Contents

Methods..**Error! Bookmark not defined.**
Methods...2
Synthesis and characterization data for anthraquinone compounds..2
Synthesis of anthraquinone-amion acid derivatives..2

Methods

Synthesis and characterization data for anthraquinone compounds

To the best of our knowledge, the synthesis of compounds has never been reported. All these target synthesized compounds were novel chemical entities characterized by IR, ^{1}H NMR, ^{13}C NMR. The results are in detail presented in the experimental part.

In the oxidation process, we tried to experiment with various oxidation systems such as chromium trioxide/sulfuricacid, chromium trioxide/glacial acetic acid, and nitric acid/nitrobenzene. In the end, we found it best to oxidize with sodium dichromate/sulfuric acid in this case. That method gave the highest product yield, and had a reaction temperature that was moderate and easy to control. During the addition of concentrated sulfuric acid, a large amount of gas bubbles were generated, which could be mitigated by the addition of a small amount of glacial acetic acid. In the acylation of 1-nitro-2-carboxy anthraquinone, the reaction was started with thionyl chloride or re-distilled Tetrahydrofuran (THF) as a solvent. After refluxing for 10 hours, the reaction was still not complete in Thin Layer Chromatography (TLC), so the reaction solvent was changed to o-dichlorobenzene. Using o-dichlorobenzene, the reaction was fully completed after refluxing at 120 ℃ for 4 hours. The reaction of 1-nitro-2-acetyl chloride anthraquinone with amino acids was done using water as a solvent. Compared to some previously reported methods, the two-phase reaction solvent system was replaced in this experiment, and the carboxyl group was not protected. However, when this experiment was carried out, it was found that pH control was particularly important. When the pH value was less than 6 or greater than 10, excessive amounts of chemical by-products were generated. Therefore, the pH value must be strictly controlled between 6-10 for this reaction.

Synthesis of anthraquinone-amion acid derivatives

Synthesis of 1-nitro-2-methylanthraquinone (2) [S1]

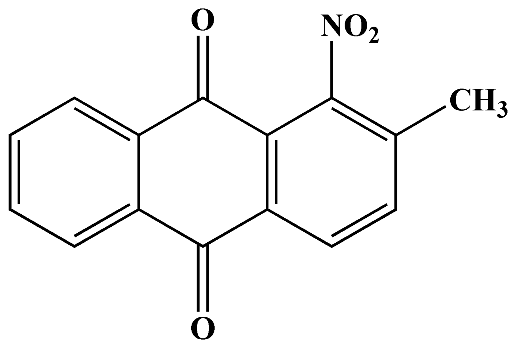

2

20 g (0.09 mol) of 2-Methylanthraquinone (1) was weighed into a 250 mL three-necked flask. 100 mL of 98% concentrated sulfuric acid was then added under cooling in an ice bath, as the mixture was allowed to below 5 ℃. 10 g of fine powder potassium nitrate powder was then added in one hour. After the addition was completed, the reaction was stirred at room temperature and the progress of the reaction was checked by TLC. After the reaction was completed, the solution was poured into 500 mL of ice water with vigorous stirring, then vacuum-filtered. The filter cake was washed first with acid water, then with hot water, and dried to obtain a crude product. This was then recrystallized from acetic acid to give a pale yellow solid of higher purity. The yield was 90.5%. Melting point: 267-269 ℃; IR, v/cm-1: 3088 (w), 3043 (w), 1680 (s), 1589 (m), 1537 (s), 1375 (w), 1328 (m), 1290 (s), 711 (m); ^{1}H NMR (300MHz, DMSO), δppm: 2.21 (s, 3H), 7.83 (d, 1H), 7.95-7.98 (m, 2H), 8.13 (d, 1H), 8.22-8.31 (m, 2H); UV λ max ($CHCl_3$) (nm): 261, 326.

Synthesis of 1-amino-2-methylanthraquinone (3)

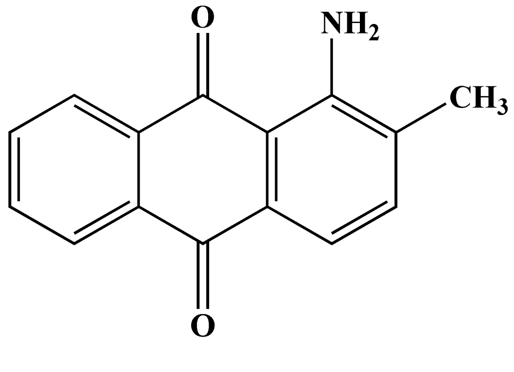

3

10.4 g (39 mmol) of compound 2 (2) was weighed into a 250 mL three-necked flask. 100 mL of

50% concentrated DMF was then added. 1.5 g (39.5 mmol) of $NaBH_4$ was then added under stirring. After the addition was completed, the reaction was stirred at room temperature and the progress of the reaction was checked by TLC. After the reaction was completed, the solution was poured into 500 mL of ice water with vigorous stirring, then vacuum-filtered. The filter cake was dried to obtain a crude product. Column chromatography was used for purification with neutral alumina. The yield was 95.6%. Melting point: 200-201 ℃; IR，v/cm^{-1}: 3423(s), 3296(m), 3068(w), 2970(w), 1658(s), 1631(m), 1608(s), 1593(m), 1552(m), 1454(m), 1380(s), 711(m); ^1H NMR (300MHz, DMSO), δ: 2.32(s,3H), 7.46(d, 1H), 7.52(d,1H), 7.92(m,2H), 8.18(d, 1H), 8.28(d, 2H); UV λ max (CH_3OH) (nm):248，309，477.

Synthesis of compound (4)

0.45 g (1.9 mmol) of 1-amino-2-methylanthraquinone was dissolved in 50 mL of anhydrous THF, and added to a 100 mL three-necked flask, 0.7 mL (5.2 mmol) of triethylamine was added. 26 mmol of acetyl chloride or chloroacetyl chloride was added dropwise in an ice bath, and the reaction was stirred at room temperature for 4 hours, and the basic reaction was completed by TLC. After the completion of the reaction, 200 mL of ethyl acetate was added, and the mixture was stirred, filtered, and the filtrate was washed twice with saturated brine and then washed with dilute sodium chloride, dried over anhydrous sodium sulfate purification (Petroleum ether: ethyl acetate = 5:1). a=acetyl chloride, b=chloroacetyl chloride

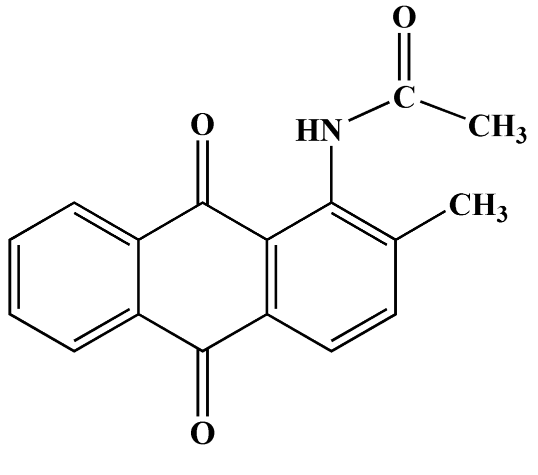

4a

4a: Melting point: 200-201 ℃; IR, v/cm^{-1}: 3430(m), 3087(w), 3024(w), 2929(w), 1670(s), 1662(s), 1589(m), 1529(m), 1328(m), 1288(s). 713(m); ^1H NMR (300MHz, DMSO), δ: 2.21(s,3H),

2.36(s,3H), 7.73-7.86(m,3H), 8.08-8.20(m, 3H), 10.00(s, 1H); UV λmax (THF) (nm):262, 335.

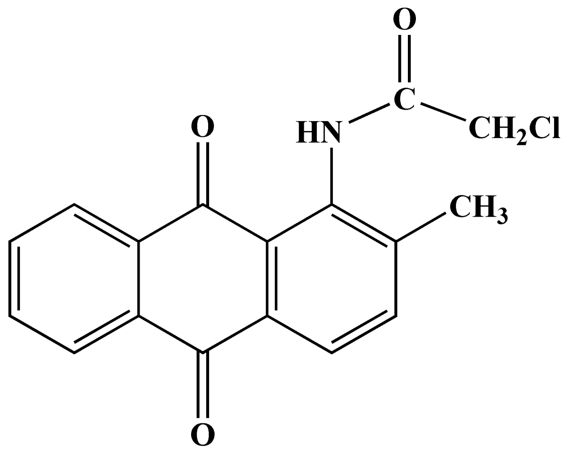

4b

4b: IR, v/cm^{-1}: 3249(w), 3024(w), 2968(w), 2935(w), 1683(s), 1666(s), 1645(m), 1589(m), 1517(s), 1469(m), 1330(m), 1286(s). 711(m); ^{1}H NMR (300MHz, DMSO), δ: 2.29(s, 3H), 4.43(s, 2H), 7.77-7.87(m, 3H), 8.05-8.09(m, 3H), 10.38(s, 1H); ^{13}C NMR (300MHz, DMSO), δ: 17.9, 44.5, 124.4, 125.4, 125.9, 131.3, 133.2, 133.5, 134.7, 135.2, 142.7, 164.0, 181.0, 182.7; UV λmax (THF) (nm): 262,335.

Synthesis of 1-nitro-2-carboxy anthraquinone (5)

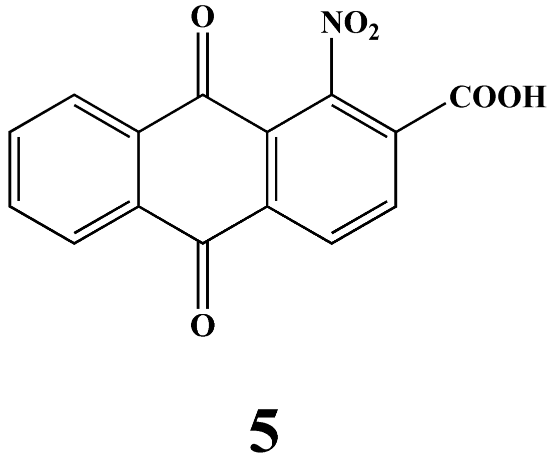

5

5.0 g (18.7 mmol) of 1-nitro-2-methylanthraquinone (2)and 15.7 g (60.0 mmol) of $Na_2Cr_2O_7$ were weighed into a 250 mL three-necked flask. 10 mL of water was added and the mixture was mixed as40 mL of 98% concentrated sulfuric acid was added under ice-water cooling. Thereaction was carried out at 60 ℃ for 5 hours before the temperature was raised to 85-90 ℃ for another 6

hours. TLC was used to detect the completion of the reaction. After cooling, the mixture was poured into a large amount of ice water while being stirred and filtered. The filter cake was then redissolved in 30 mL ofa 5% NaOH aqueous solution at 90 ℃. After dissolving for 1 hour and undergoing another round of vacuum-filtration, the filtrate was acidified with 6 mol/L hydrochloric acid, vacuum filtered, and the filter cake was dried to obtain 3.2 g of a solid crystal. The yield was 57.5%. Melting point: 286 ℃; IR, v/cm-1: 3595 (w), 3525 (w), 3093-3083 (w), 1706 (s), 1685 (s), 1589 (m), 1554 (s), 1469 (m), 1371 (w), 1317 (m), 1276 (s), 707 (m); UV λ max (THF) (nm): 260, 326.

Synthesis of 1-nitro-2-benzoyl anthraquinone (6) [S2]

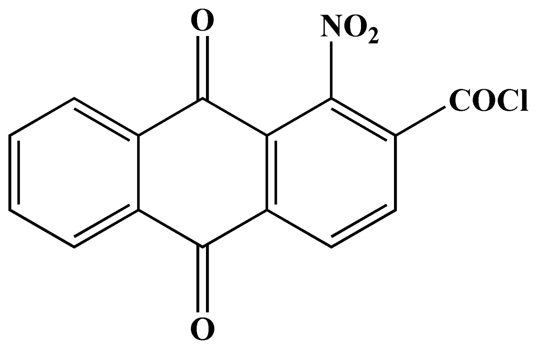

6

3.5 g (11.8 mmol) of 1-nitro-2-carboxy anthraquinone (3) was weighed into a 100 mL three-necked flask. 40 mL of chlorobenzene was added as a solvent. 5 mL of resteamed $SOCl_2$ at room temperature was then added. The mixture was heated to 120 ℃, and refluxed for 4 hours. After the reaction was completed, it was cooled to room temperature and vacuum-filtered. The filter cake was washed with a small amount of anhydrous diethyl ether and dried under an infrared lamp to obtain 3.2 g of beige solid. The yield was 57.5%. The product was directly subjected to the next reaction without further purification. Melting point: 286 ℃.

Synthesis of 1-amino-2-carboxy anthraquinone (7)

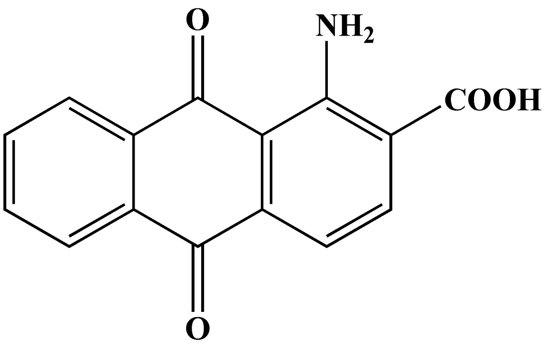

7

6.0 g (20.2mmol) of 1-nitro-2-carboxy anthraquinone (5) was weighed into a 500mL single-necked flask, add 400mL of 1% ammonia solution as solvent, and slowly add 10mL of 0.7g (18.5mmol) of $NaBH_4$ aqueous solution at room temperature. After the addition is completed, the reaction solution is allowed to stand at room temperature. After stirring for 4 hours, TLC followed the progress of the reaction. After completion of the reaction, the reaction solution was diluted with 100 mL of distilled water, acidified with dilute hydrochloric acid, suction filtered, and the filter cake was washed three times with water and dried to give a product of 5.1 g. The yield was 99%.

General synthesis of anthraquinone-amino acid compounds (8)（8a-8i）

About 7.5 mmol of each amino acid was weighed into a 100 mL three-necked flask. 25 mL of an aqueous solution containing about 0.3 g of NaOH was added, the mixture was stirred and cooled to 0 ℃ with ice water, and 1.6 g of 1-nitro-2-benzoyl anthraquinone (4) (5 mmol) in THF was added along with a 5 mL of an NaOH solution after 20 min. A dropping funnel was used to maintain a pH of 8-10 in the vigorously stirred solution. After completion of the dropwise addition, the reaction continued under ice water for 1 hour, and the reaction was allowed to proceed overnight at room temperature. The following day, the reaction mixture was poured into 200 mL of ice water, acidified with dilute hydrochloric acid (pH 1-2), stirred, vacuum-filtered, and the filtrate was washed with a small amount of water several times to obtain a solid product. This product was then separated and purified by column chromatography.

N-[(9,10-dihydro-1-nitro-9,10-dioxo-2-anthracenyl)carbonyl-Valine] (**8b**)

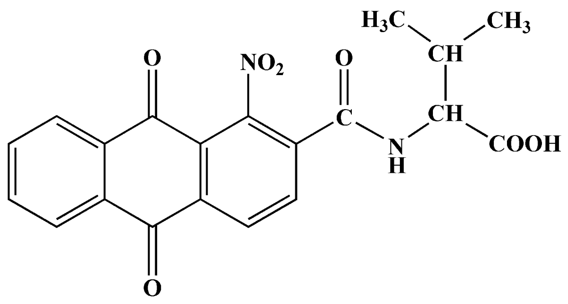

8b

IR, v/cm-1: 3421 (w), 3500-2500 (w), 3076 (w), 2966 (w), 1720 (w), 1681 (s), 1591 (m), 1550 (s), 1467 (w), 1371 (m), 1286 (m), 1234 (w), 711 (m); ^{1}H NMR (300 MHz, DMSO), δppm: 0.92 (d, 6H), 2.15 (m, 1H) , 4.26 (m, 1H), 7.93 (m, 2H), 8.08-8.20 (m, 3H), 8.44 (d, 1H), 9.23 (d, 1H), 12.85 (s, 1H); ^{13}C NMR (300 MHz, DMSO), δppm: 20.3, 21.3, 31.9, 60.2, 129.1, 130.9, 134.7, 135.3, 135.6, 136.8, 137.1, 137.4, 148.1, 165.8, 174.5, 182.1, 182.7; UV λ max (THF) (nm): 260 , 326.

N-[(9,10-dihydro-1-nitro-9,10-dioxo-2-anthracenyl)carbonyl-Phenylalanine] (**8c**)

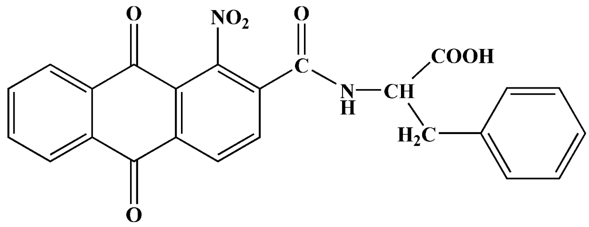

8c

Melting point > 250 ℃; IR，v/cm-1: 3063 (w), 1710 (m), 1684 (s), 1632 (s), 1590 (m), 1545 (s), 713 (m);^{1}H NMR (300 MHz, DMSO), δppm: 7.92 (d, 2H), 8.20-8.45 (m, 6H); UV λ max (THF) (nm): 263, 326.

N-[(9,10-dihydro-1-nitro-9,10-dioxo-2-anthracenyl)carbonyl-Glycine (**8d)**

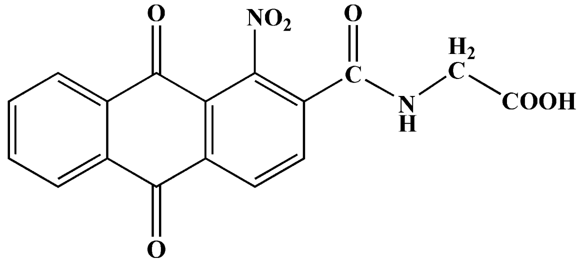

8d

IR, v/cm-1: 3380 (w), 3303 (w), 3500-2500 (m), 1743 (m), 1685 (s), 1656 (s), 1589 (m), 1554

(s), 1413 (w), 1321 (m), 1280 (s), 709 (m); ^{1}H NMR (300 MHz, DMSO), δppm: 3.96 (d, 2H), 7.95-7.98 (m, 2H), 8.13-8.22 (m, 2H), 8.50 (d, 1H), 9.41(s, 1H), 12.80 (s, 1H); ^{13}C NMR (300 MHz, DMSO), δppm: 45.4, 128.6, 131.1, 131.2, 133.1, 136.9, 137.4, 138.4, 139.2, 139.7, 150.4, 167.6, 174.8, 184.0, 184.7; UV λ max (THF) (nm): 260, 326.

N-[(9,10-dihydro-1-nitro-9,10-dioxo-2-anthracenyl)carbonyl-Proline] (**8e**)

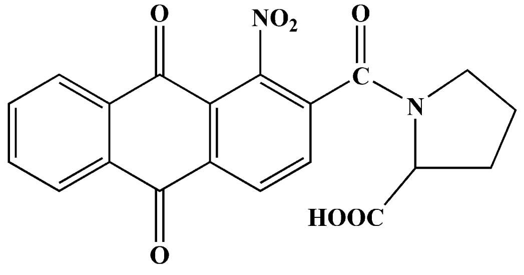

8e

IR, ν/cm-1: 3500-2500(w), 3069(w), 1720(m), 1678(s), 1635(s), 1590(m), 1546(s), 713(m); ^{1}H NMR (300 MHz, DMSO), δppm: 3.00(m, 1H), 3.18(m, 1H), 4.56(s, 1H), 7.22-7.29(m, 5 H), 7.95-8.46(m, 6H), 9.42(s, 1H), 12.96(s, 1H); ^{13}C NMR (300 MHz, DMSO), δppm: 38.2, 56.0, 125.7, 128.5, 128.9, 130.3, 130.7, 131.0, 134.7, 135.2, 136.0, 136.9, 139.5, 148.0, 164.9, 174.2, 181.8, 182.5; UV λ max (THF) (nm): 263, 326.

N-[(9,10-dihydro-1-nitro-9,10-dioxo-2-anthracenyl)carbonyl-Alanine] (**8f**)

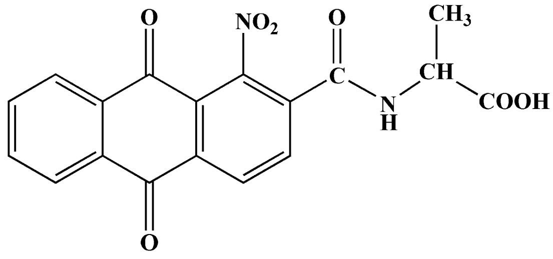

8f

IR, ν/cm-1: 3365(w), 3500-2500(m), 1731(m),1675(s), 1593(m), 1552(s), 1411(w), 709(m); ^{1}H NMR (300MHz, DMSO), δppm: 1.36(d, 3H), 4.35(m, 1H), 7.92-8.49(m, 6H), 9.41(d,1H), 12.73(s, 1H); UV λ max (THF) (nm): 260,326

N-[(9,10-dihydro-1-nitro-9,10-dioxo-2-anthracenyl)carbonyl-methionine] (**8g**)

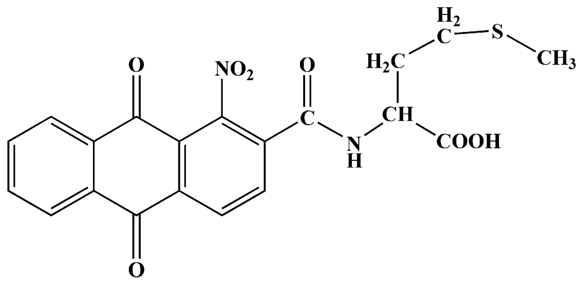

8g

IR, v/cm-1: 3365(w), 3500-2500(m), 1731(m),1675(s), 1593(m), 1552(s), 1411(w), 709(m); ^{1}H NMR (300MHz, DMSO), δppm: 1.36(d, 3H), 4.35(m, 1H), 7.92-8.49(m, 6H), 9.41(d,1H), 12.73(s, 1H); UV λ max (THF) (nm): 260,326

N-[(9,10-dihydro-1-nitro-9,10-dioxo-2-anthracenyl)carbonyl-methionine] (**8g**)

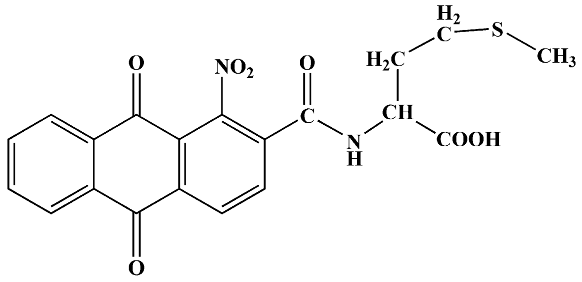

8g

IR, v/cm-1: 3487(w), 3371(w), 3500-2500(w), 2960(w), 1724(m), 1680(s), 1658(s), 1589(m), 1552(s), 1469(w), 1411(w), 1369(m), 1319(m), 1280(s), 711(m); ^{1}H NMR (300MHz,DMSO), δppm: 2.08-2.11(m, 1H), 2.24(m, 1H), 2.58(s, 1H), 2.80-2.88(m, 3H), 4.49(s, 1H), 7.97-7.99(m, 2H), 8.13-8.24(m, 3H), 8.48-8.51(d,1H), 9.48-9.51(d,1H), 13.10(s, 1H); UV λ max (THF) (nm): 261, 326.

N-[(9,10-dihydro-1-nitro-9,10-dioxo-2-anthracenyl)carbonyl-Glutamic acid] (**8h**)

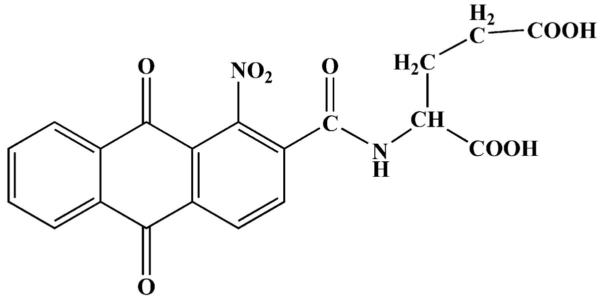

8h

^{1}H NMR (300MHz,DMSO), δppm: 1.92(m, 1H), 2.08(m, 1H), 2.36(m, 2H), 4.34(d, 1H),

7.97-8.45(m, 6H), 9.20(s, 1H); ^{13}C NMR (300MHz,DMSO), δppm: 26.7, 30.7, 52.7, 124.6, 127.1, 127.2, 129.1, 132.8, 133.4, 134.6, 135.3, 135.6, 146.3, 163.3, 173.2, 174.3, 180.2, 180.8.

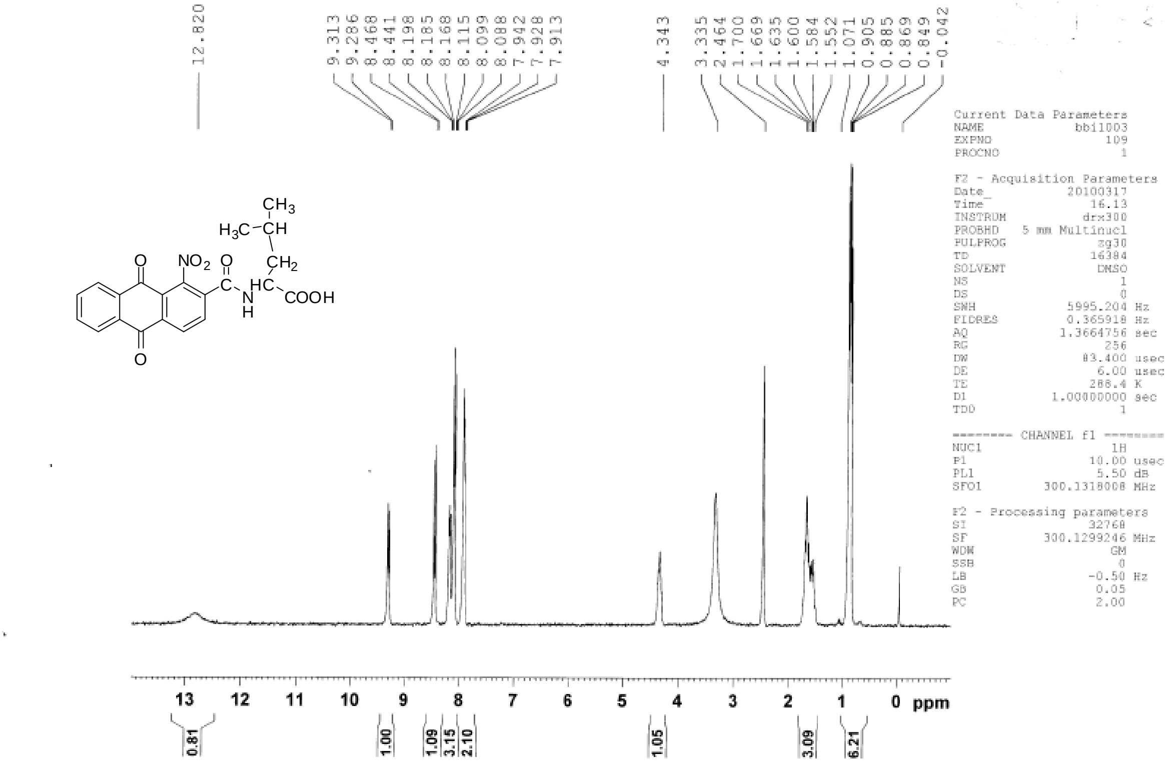

Figure S1. The ^{1}HNMR (300 MHz) spectrum of compound **8a**

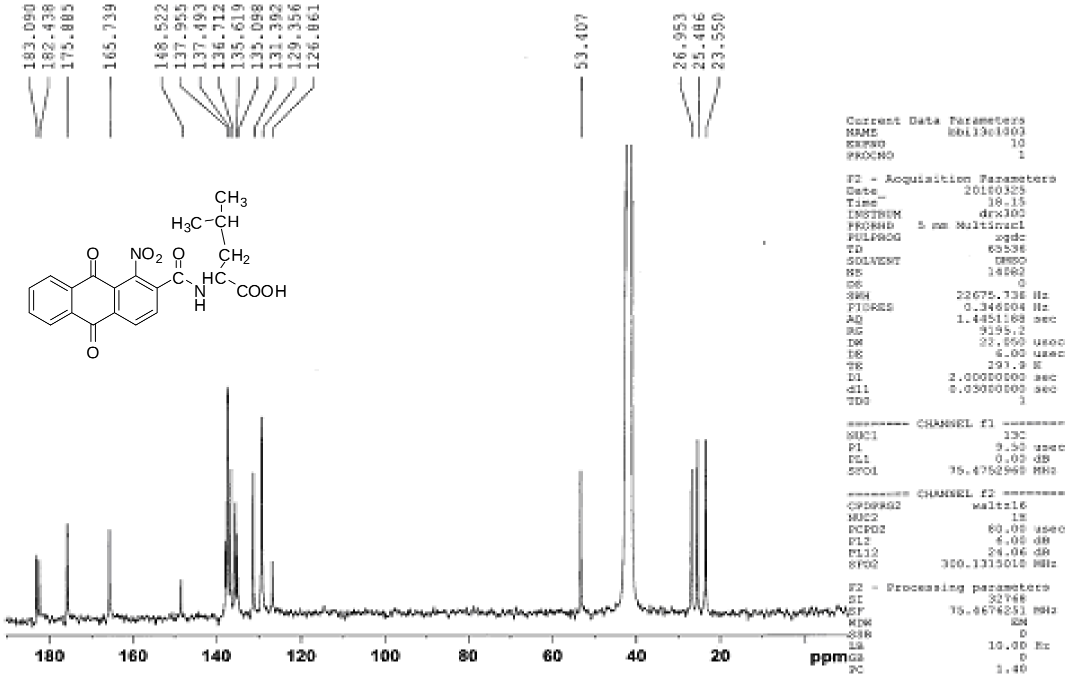

Figure S2. The ^{13}CNMR (300 MHz) spectrum of compound **8a**

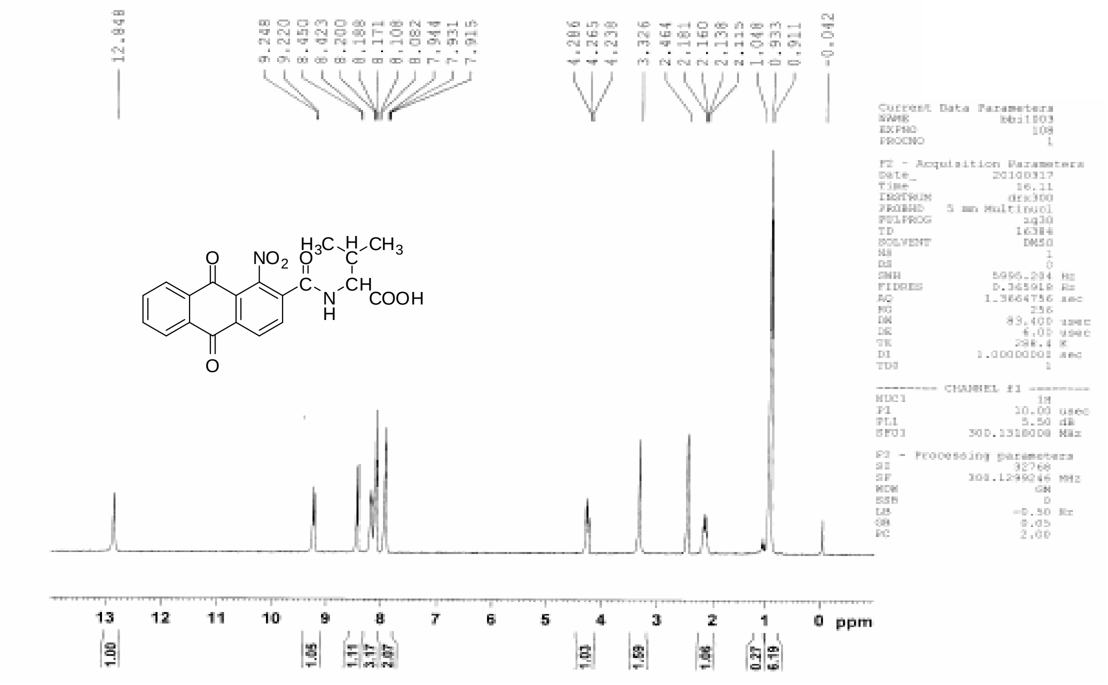

Figure S3. The ^{1}HNMR (300 MHz) spectrum of compound **8b**

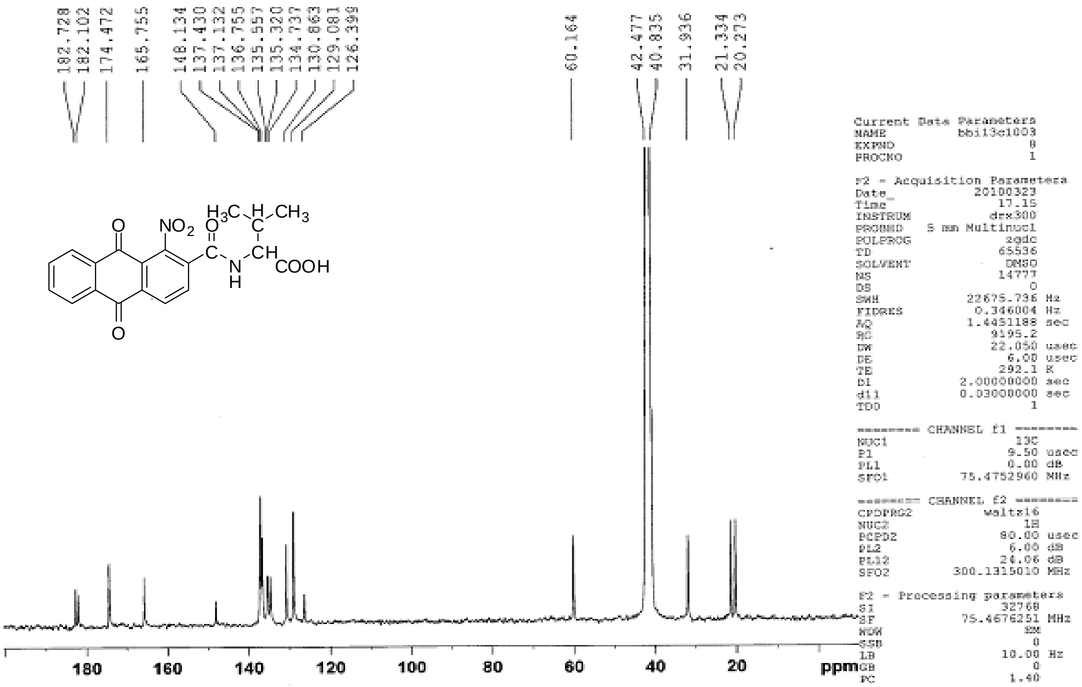

Figure S4. The ^{13}CNMR (300 MHz) spectrum of compound **8b**

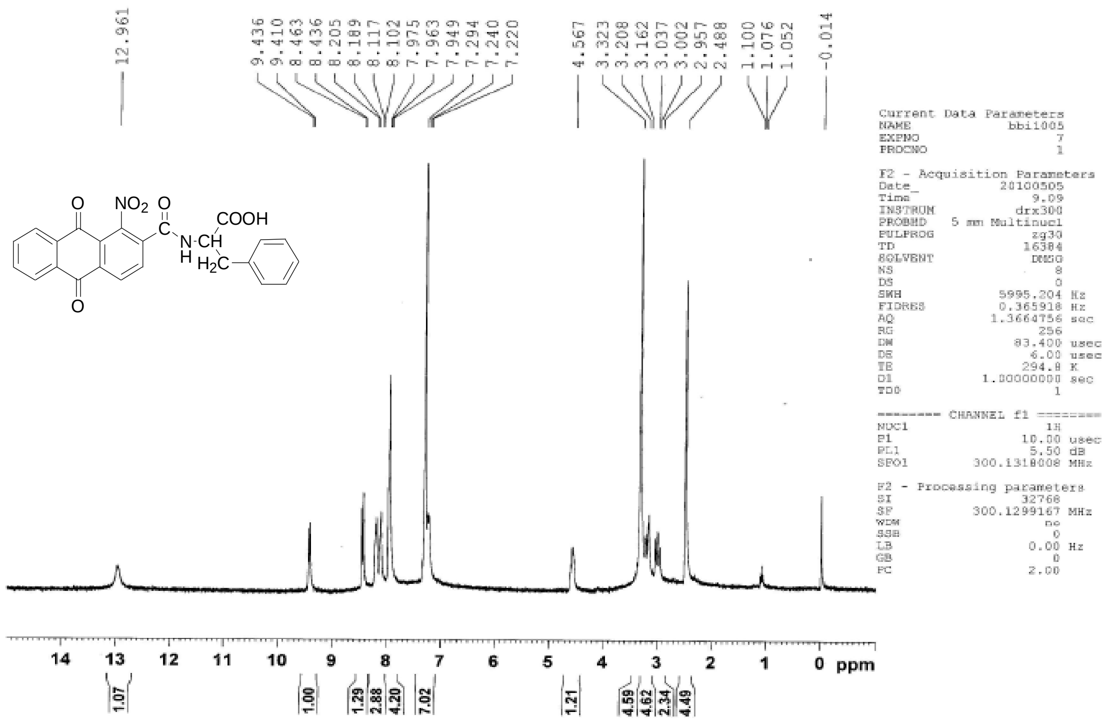

Figure S5. The ^{1}HNMR (300 MHz) spectrum of compound **8c**

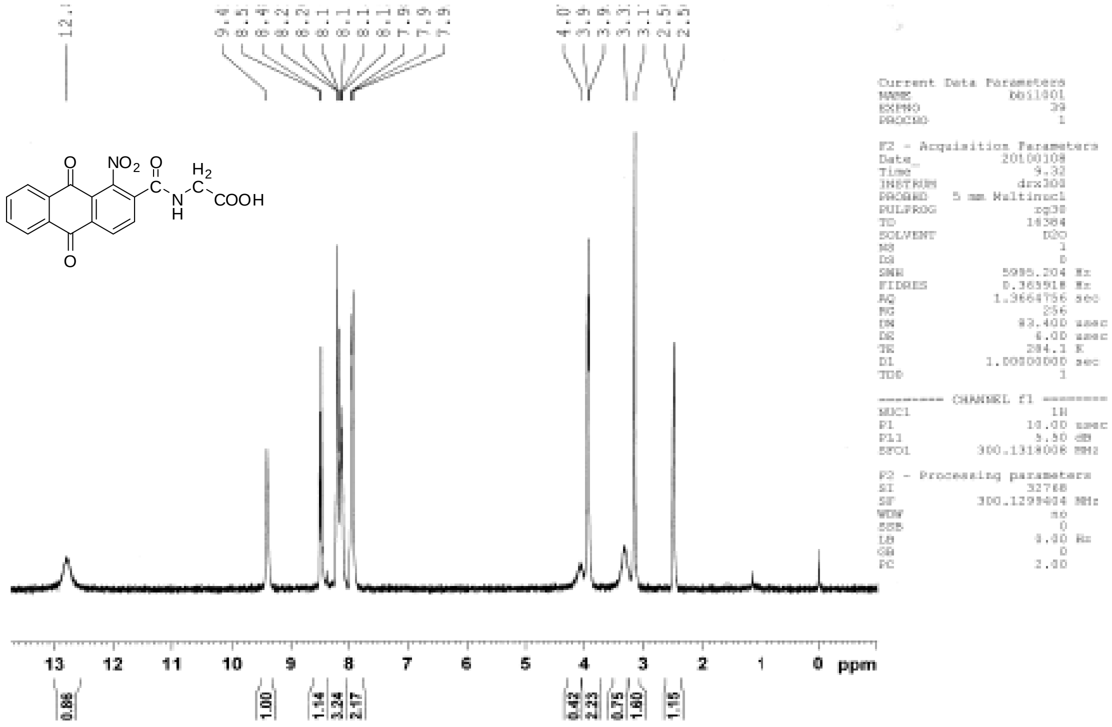

Figure S6. The ^{1}HNMR (300 MHz) spectrum of compound **8d**

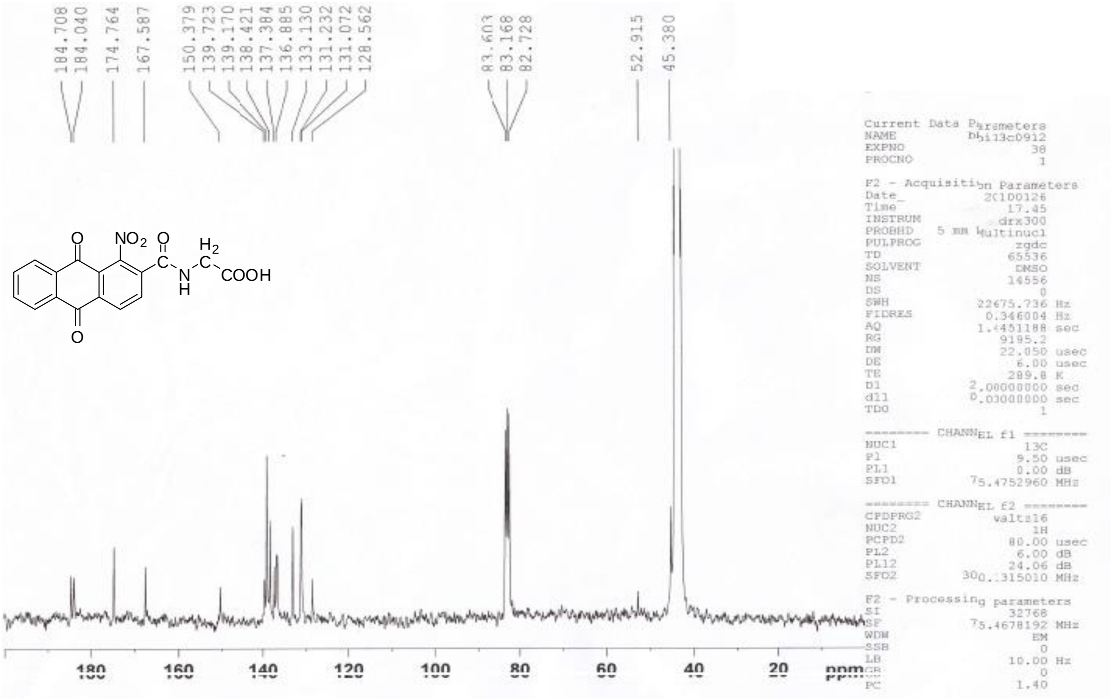

Figure S7. The ^{13}CNMR (300 MHz) spectrum of compound **8d**

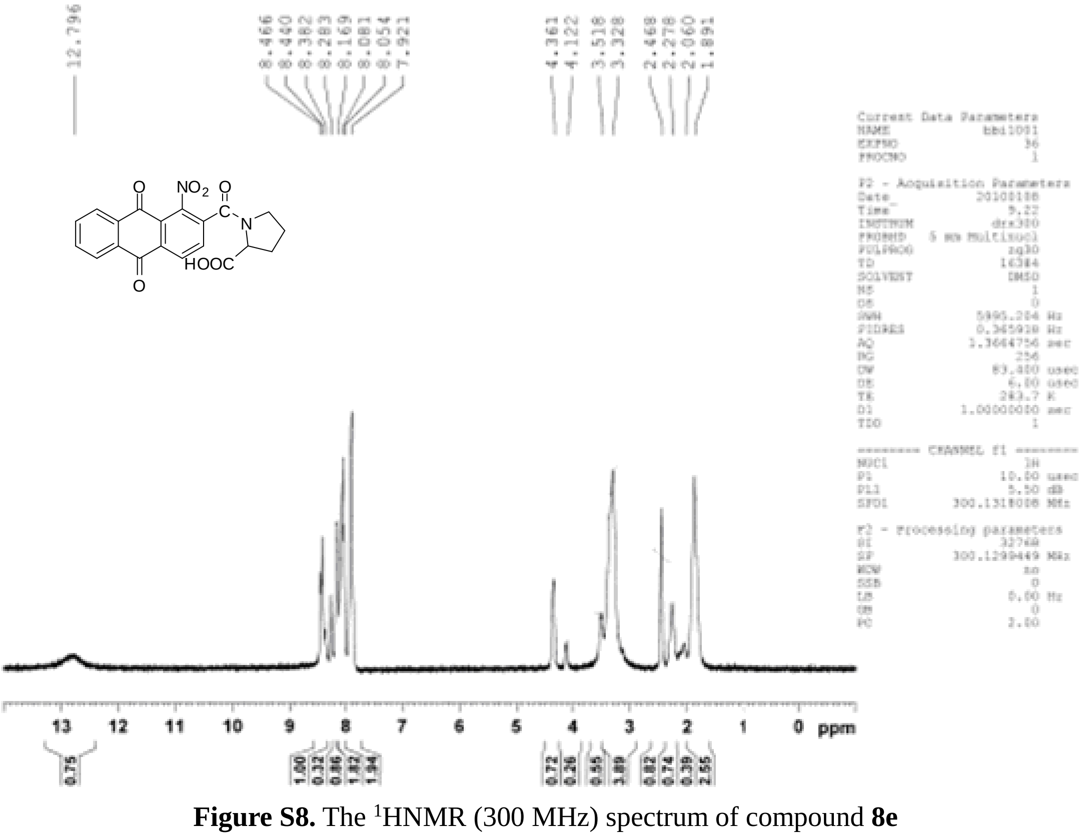

Figure S8. The ^{1}HNMR (300 MHz) spectrum of compound **8e**

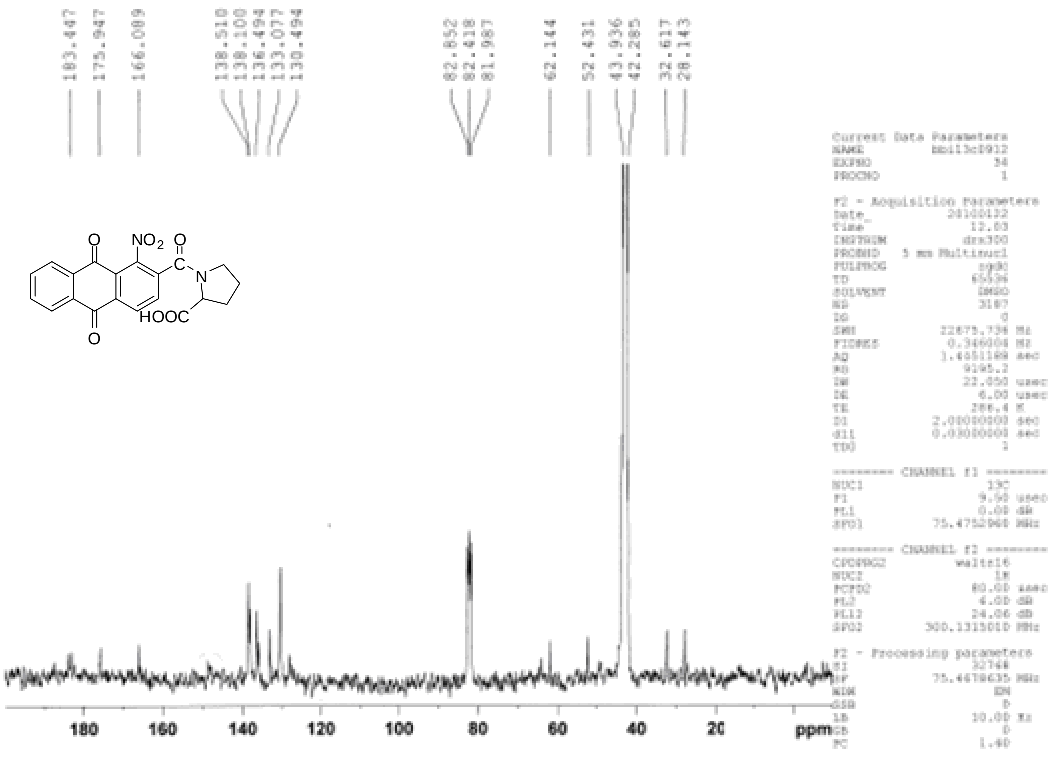

Figure S9. The ^{13}CNMR (300 MHz) spectrum of compound **8e**

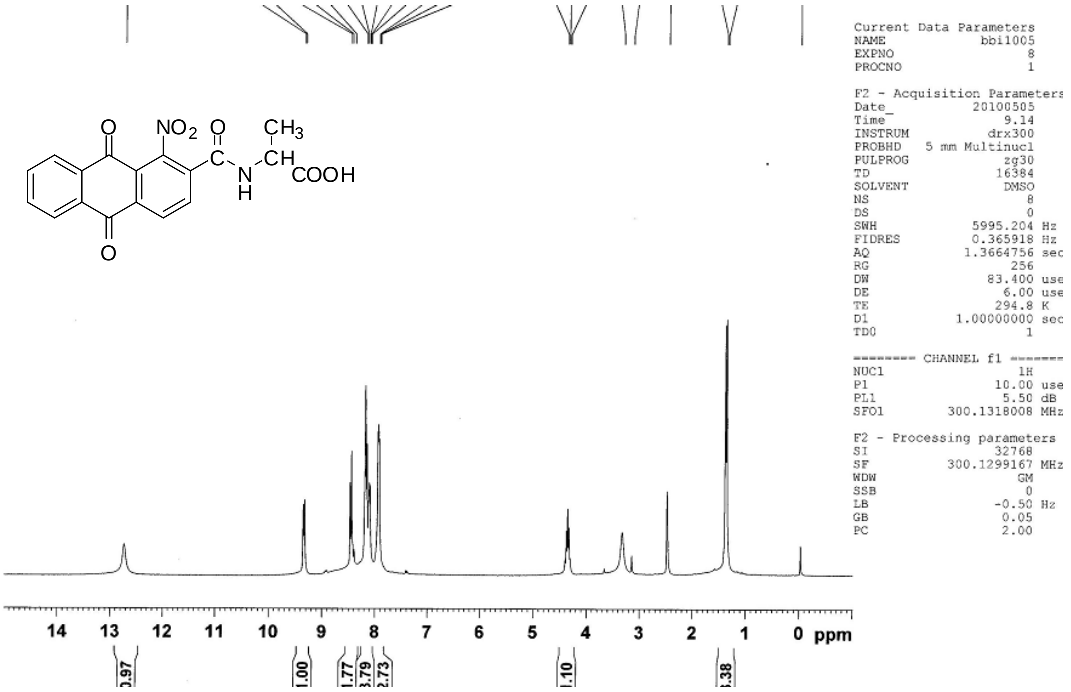

Figure S10. The ^{1}HNMR (300 MHz) spectrum of compound **8f**

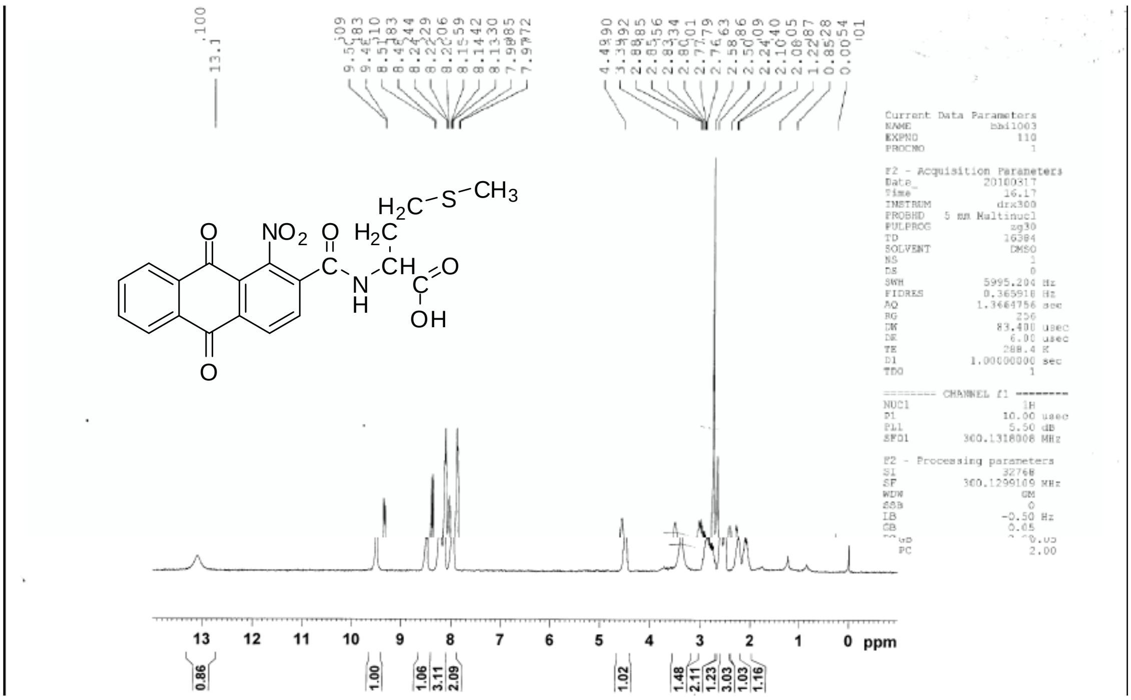

Figure S11. The ^{1}HNMR (300 MHz) spectrum of compound **8g**

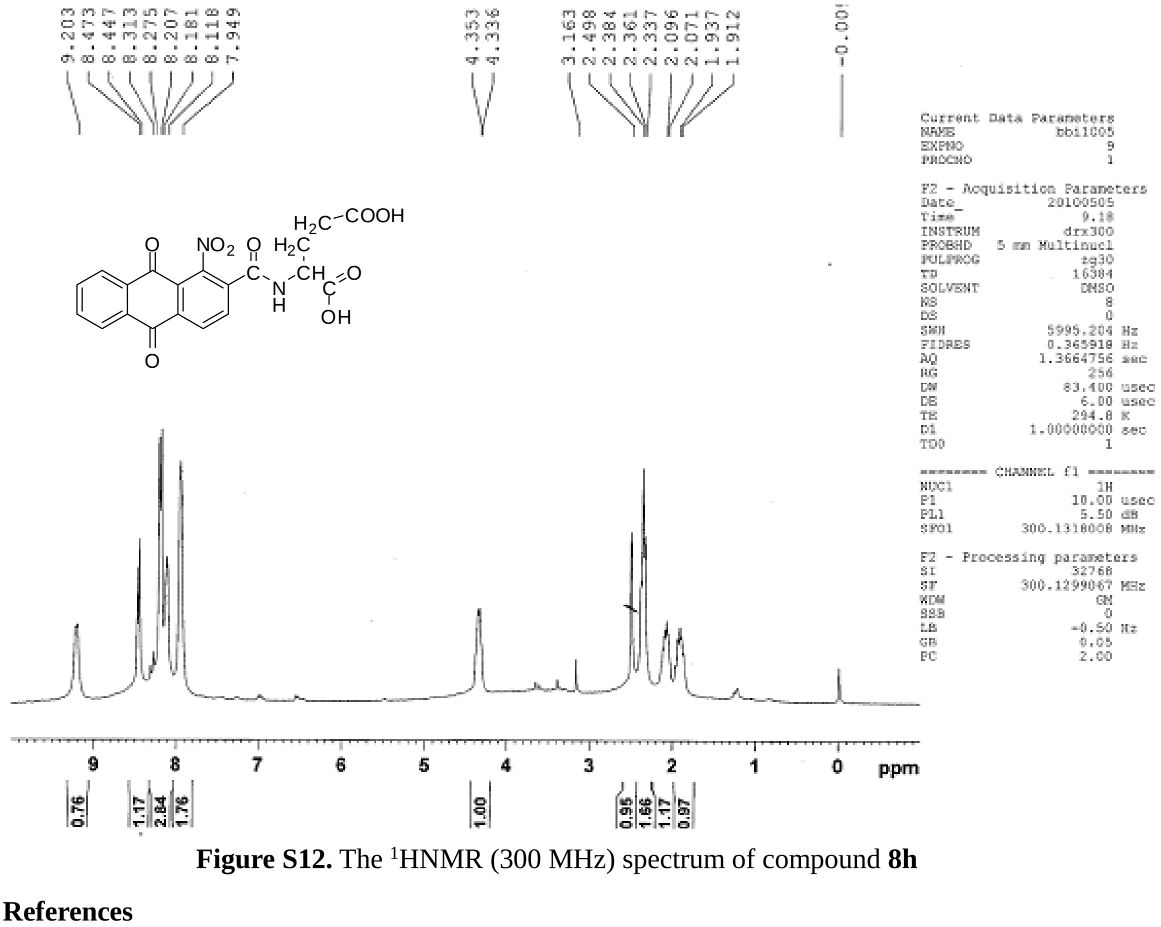

Figure S12. The ^{1}HNMR (300 MHz) spectrum of compound **8h**

References

[S1] Wood, G.D; Peters, A.T; 1962. Reactions of 2-methyl-1-nitro-and 1-amino-2-methyl-anthraquinone derived dyes for cellulose acetate rayon. Journal of the Chemical Society. 667, 3373-3378.

[S2] Morley, J. O. 1976. Synthesis of aminoanthraquinones by sodium boro-hydride reductions of nitroanthraquinones. Chemischer Informationsdienst. 7, 249-251.
